# TAp63γ is the primary isoform of TP63 for tumor suppression but not development

**DOI:** 10.1038/s41420-025-02326-x

**Published:** 2025-02-06

**Authors:** Xinbin Chen, Wenqiang Sun, Xiangmudong Kong, Xin Ming, Yanhong Zhang, Wensheng Yan, Shakur Mohibi, Mingyi Chen, Keith Mitchell, Jin Zhang

**Affiliations:** 1 Comparative Oncology Laboratory, Schools of Veterinary Medicine and Medicine, University of California, Davis, USA; 2 Department of Pathology, University of Texas Southwestern Medical Center, Dallas, Texas 75390 USA; 3 Department of Physiology and Membrane Biology, University of California Davis School of Medicine, Davis, Davis, CA USA; 4 Present Address: Department of Animal Science and Technology, Sichuan Agricultural University, Ya’an, China

**Keywords:** Cell biology, Tumour-suppressor proteins, Cell growth

## Abstract

TP63 is expressed as TAp63 and ΔNp63 from the P1 and P2 promoters, respectively. While TAp63 and ΔNp63 are expressed as three TAp63α/β/γ and ΔNp63α/β/γ due to alternative splicing, only p63α (TA and ΔN) and p63γ (TA and ΔN) proteins are found to be detectable and likely to be responsible for p63-dependent activity. Previous studies implied and/or demonstrated that TAp63α, which contains an N-terminal activation domain conserved in p53, functions as a tumor suppressor by regulating an array of genes for growth suppression. By contrast, ΔNp63α, which also contains an N-terminal activation domain but is different from that in TAp63, regulates a unique set of genes and functions as a master regulator for development of epidermis and other stratified epithelial tissues. However, the biological function of p63γ is largely unexplored. To explore this, we generated a mouse model in that exon 10’, a coding exon specific for p63γ, was deleted by CRISPR-cas9. We showed that mice deficient in *p63*γ are viable and futile, which is different from mice deficient in total TP63 or p63α. Like TAp63-deficient mice, p63γ-deficient mice have a short lifespan and are prone to spontanenous tumors. Additionally, loss of p63γ shortens the lifespan of tumor-free mice potentially via increased cellular senescence. Moreover, mice deficient in *p63*γ are prone to chronic inflammation in multiple organs and liver steatosis potentially via altered lipid metabolism. Single-cell RNA-seq revealed that loss of p63γ increases the expression of SCD1, a rate-limiting enzyme for synthesis of monounsaturated fatty acids, leading to altered lipid homeostasis. Together, our data indicate that TP63γ is the primary isoform of TP63 for tumor suppression but not development by maintaining normal inflammatory response and lipid homeostasis.

## Introduction

**Fig. 1:**
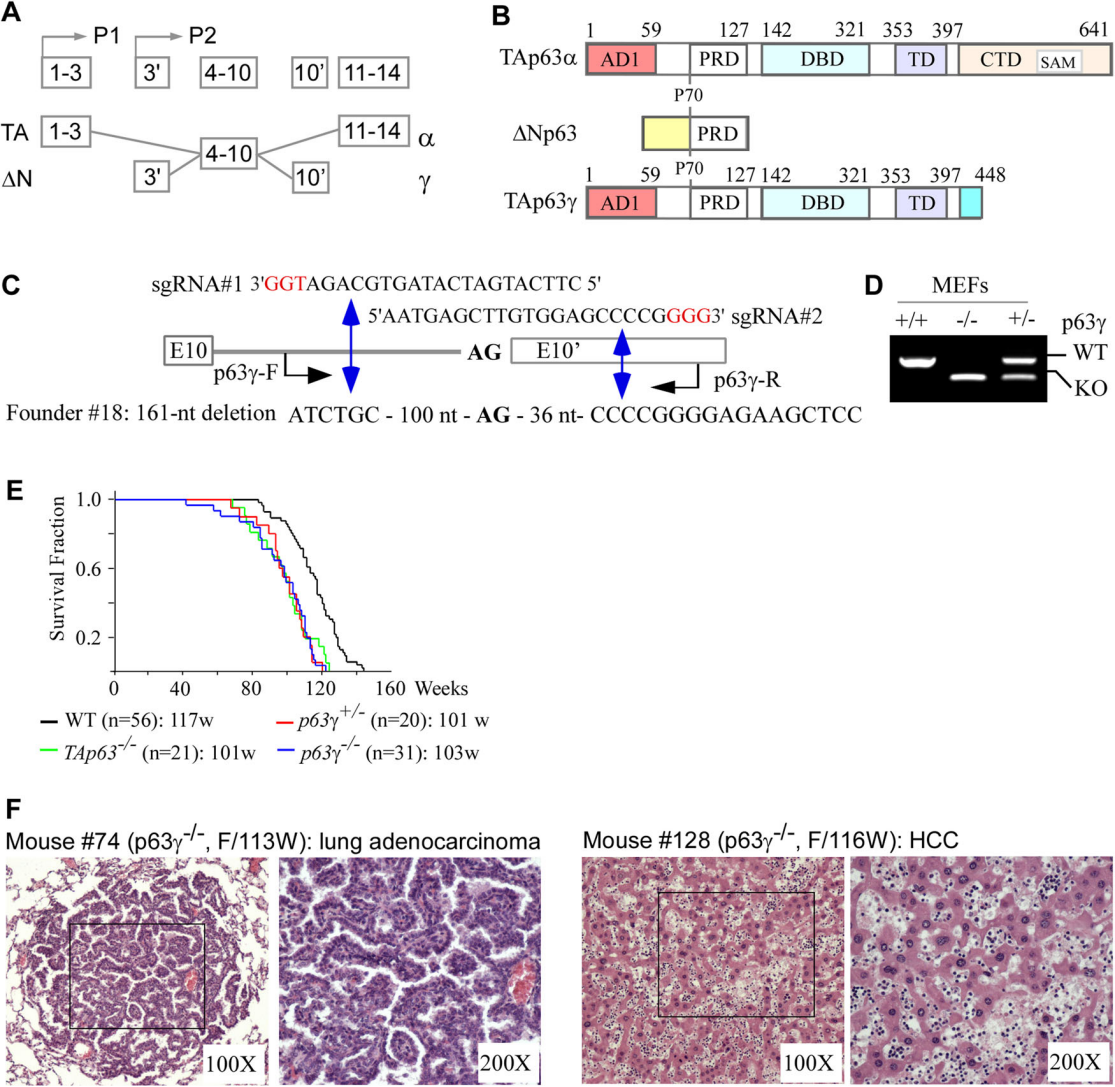
Mice deficient in p63*γ* have a short lifespan and are prone to spontaneous tumors. **A** The TP63 gene locus, the P1 promoter for TAp63, and the P2 promoter for ΔNp63. **B** The structure and functional domains of TAp63α, ΔNp63, and TAp63γ proteins. AD: activation domain. PRD: proline-rich domain. DBD: DNA-binding domain. TD: tetramerization domain. SAM: sterile alpha motif. **C** The strategy to knock out p63γ-specific exon 10’. The location and sequence of the two sgRNAs and the location of p63γ-F/R primers are indicated. The predicted double-strand breaks near the PAM motifs (in red) are indicated by double blue arrow. Founder #18 has a deletion of 161 nucleotides spanning from intron 10 to exon 10’, including the splice acceptor site (AG). **D** Genotyping of wild-type and p63g-null alleles of Wild-type, *p63γ*^*+/−*^, and *p63γ*^*−/−*^ MEFs. **E** Kaplan–Meier survival curves of WT (*n* = 56), *TAp63*^*+/−*^ (*n* = 21), *p63γ*^*+/−*^ (*n* = 20), and *p63γ*^*−/−*^ (*n* = 33) mice. **F** Representative images of H.E.-stained lung adenocarcinoma in p63γ-null mouse #74 and hepatocellular carcinoma in p63γ-null mouse #128.

The *p63* gene is expressed as TAp63 from the P1 promoter and as ΔNp63 from the P2 promoter (Fig. 1A). Through alternative splicing and transcriptional termination, at least three TA and three ΔN isoforms (α−γ are expressed and can be detected by RT-PCR in some tissues [1–3]. TAp63 contains an N-terminal activation domain conserved in p53 (Fig. 1B) and functions as a tumor suppressor by regulating an array of genes for growth suppression [4–6]. ΔNp63 also contains an N-terminal activation domain, which is different from the activation domain in TAp63, regulates a unique set of genes and functions as a master regulator for development of epidermis and other stratified epithelial tissues [7–9]. Among the C-terminal isoforms, TAp63α and TAp63γ share identical activation domain (AD), proline-rich domain (PRD), DNA-binding domain (DBD), and tetramerization domain (TD) (Fig. 1B). However, TAp63α has 231 unique amino acids in its C-terminus, which contains the C-terminal inhibitory domain, including the sterile alpha motif (Fig. 1B). TAp63γ has 38 unique amino acids with unknown activities (Fig. 1B). Studies showed that these unique C-terminal sequences can confer activities specific to TAp63α and TAp63γ by regulating a different set of targets [10–13].

Lipids represent a complex group of biomolecules, including cholesterol and cholesterol esters (CEs), triglycerides (TAGs) and phospholipids (such as phosphatidylcholine (PC), phosphatidylethanolamine (PE) and phosphatidylserine (PS)). Lipids are required for the maintenance of cellular structures, energy supply, and diverse aspects of signal transduction. Studies have shown that cancer cells exhibit specific alterations in lipid homeostasis, such as increase in lipogenesis and lipid uptake and storage [14, 15]. These alterations are largely due to elevated expression or activities of lipogenic transcription factors, especially the Sterol Regulatory Element-Binding Proteins (SREBPs). SREBPs are ER-bound transcription factors and expressed as three isoforms: SREBP-1a and -1c, which are produced from the SREBF1 gene through utilization of alternative promoters, and SREBP-2, which is encoded by the SREBF2 gene. SREBP-2 preferentially regulates genes responsible for cholesterol synthetic pathway. SREBP-1c is primarily control genes involved in synthesis of fatty acids (FAs) and TAGs. SREBP-1a have overlapping functions with both SREBP-1c and SREBP-2. Notably, elevated expression of SREBPs is associated with tumor progression and poor prognosis in many cancers [16].

Previous studies implied and/or demonstrated that TAp63α functions as a tumor suppressor whereas ΔNp63α functions as a master regulator for development of epidermis and other stratified epithelial tissues. However, the biological function of p63γ is largely unexplored. Here, we showed that mice deficient in *p63*γ are viable and fertile but prone to spontanenous tumors with a short lifespan. Additionally, loss of p63γ shortens the lifespan of tumor-free mice potentially via increased cellular senescence. Moreover, mice deficient in *p63*γ are prone to chronic inflammation and liver steatosis potentially via altered lipid metabolism. Furthermore, loss of p63γ increases the expression of SCD1, a rate-limiting enzyme for synthesis of monounsaturated fatty acids, leading to altered lipid homeostasis. Together, our data indicate that TP63γ is the primary isoform of TP63 for tumor suppression but not development by maintaining normal inflammatory response and lipid homeostasis, which can be explored as a therapeutic strategy to kill p63γ-deficient tumors.

## Results

### Mice deficient in *p63*γ have a short lifespan and are prone to spontanenous tumors

To generation of a p63γ isoform-specific knockout mouse model, p63γ-specific exon 10’ was deleted by CRISPR-cas9 with one sgRNA targeting intron 10 and the other targeting exon 10’ (Fig. 1C). Three founder mice were generated by CRISPR-Cas9 and genotyped by sequencing. Founder #18 has a 161-nt deletion spanning the intron 10 and exon 10’, including the splice acceptor site (AG) (Fig. 1C). Due to lack of the splicing acceptor site (AG) and the coding sequence in exon 10’, Founder #18 mouse would be deficient in p63γ. Using Founder #18, we generated a cohort of WT, *p63γ*-het and *p63γ*-null MEFs, which had predicted wild-type and KO alleles of p63γ (Fig. 1D).

Since CRISPR knockout may have an off-target deletion, Founder #18 mice were crossed with C57BL/6 mice to breed out any potential off-target deletion as long as the off-target deletion is not located on the same chromosome as the p63 gene (chromosome 16). Next, *p63γ*^*+/−*^ mice were intercrossed to generate a cohort of *p63γ*^*+/−*^ and *p63γ*^*−/−*^ mice, which were monitored throughout their lifespan. Previous studies showed that *Trp63*^*−/−*^, *p63*α^*−/−*^ and Δ*Np63*^*−/−*^ mice were not viable and/or died soon after birth whereas *TAp63*^*−/−*^ mice are viable [7, 17–19]. Here, we found that *p63γ*^*−/−*^ mice were viable and fertile, suggesting that both TAp63γ and ΔNp63γ are not required for embryonic development and reproduction. We also found that simiar to *TAp63*^*+/−*^ mice, both *p63*γ^*+/−*^ and *p63*γ^−/−^ mice had a short lifespan as compared to wild-type mice (Fig. 1E and Supplementary table S1–4). The mean lifespan was 101 weeks for *p63*γ^*+/−*^ mice and 103 weeks for *p63*γ^−/−^ mice, which were significantly shorter than 117 weeks for wild-type mice (*p* < 0.01 by logRank test (WT vs *p63*γ^+/−^; WT vs *p63*γ^−/−^). Moreover, we found that like *TAp63*^*+/−*^ mice, both *p63*γ^*+/−*^ and *p63*γ^−/−^ mice were prone to spontaneous tumors (Table 1; Fig. 1F). The tumor spectra were very similar in *TAp63*^*+/−*^, *p63*γ^*+/−*^ and *p63*γ^−/−^ mice, with lymphomas and sarcomas as the most common types (Fig. 1F). We also noticed that both *p63*γ^*+/−*^ and *p63*γ^−/−^ mice developed hepatocellular carcinoma (HCC) whereas *TAp63*^*+/−*^ mice did not (Fig. 1F, G).

**Table 1:** Tumor spectra for WT, *TAp63*^*+/−*^, *p63γ*^*+/−*^, and *p63γ*^*−/−*^ mice.

Tumor type	WT (*n* = 51)	*TAp63*^*+/−*^ (*n* = 21)	*p63γ*^*+/−*^ (*n* = 20)	*p63γ*^*−/−*^ (*n* = 31)
Lymphoma	11	5	6	11
Sarcoma	1	8	1	2
HCC	0	0	1	2
Adenocarcinoma	0	0	1	1
Adenoma	0	0	1	0
Hemangioma	0	1	0	0
Infarcted hematoma	0	1	1	0
Tumor penetrance	11/51	12/21	11/20	14/31

### Loss of p63γ shortens the lifespan of tumor-free mice potentially via increased cellular senescence

**Fig. 2:**
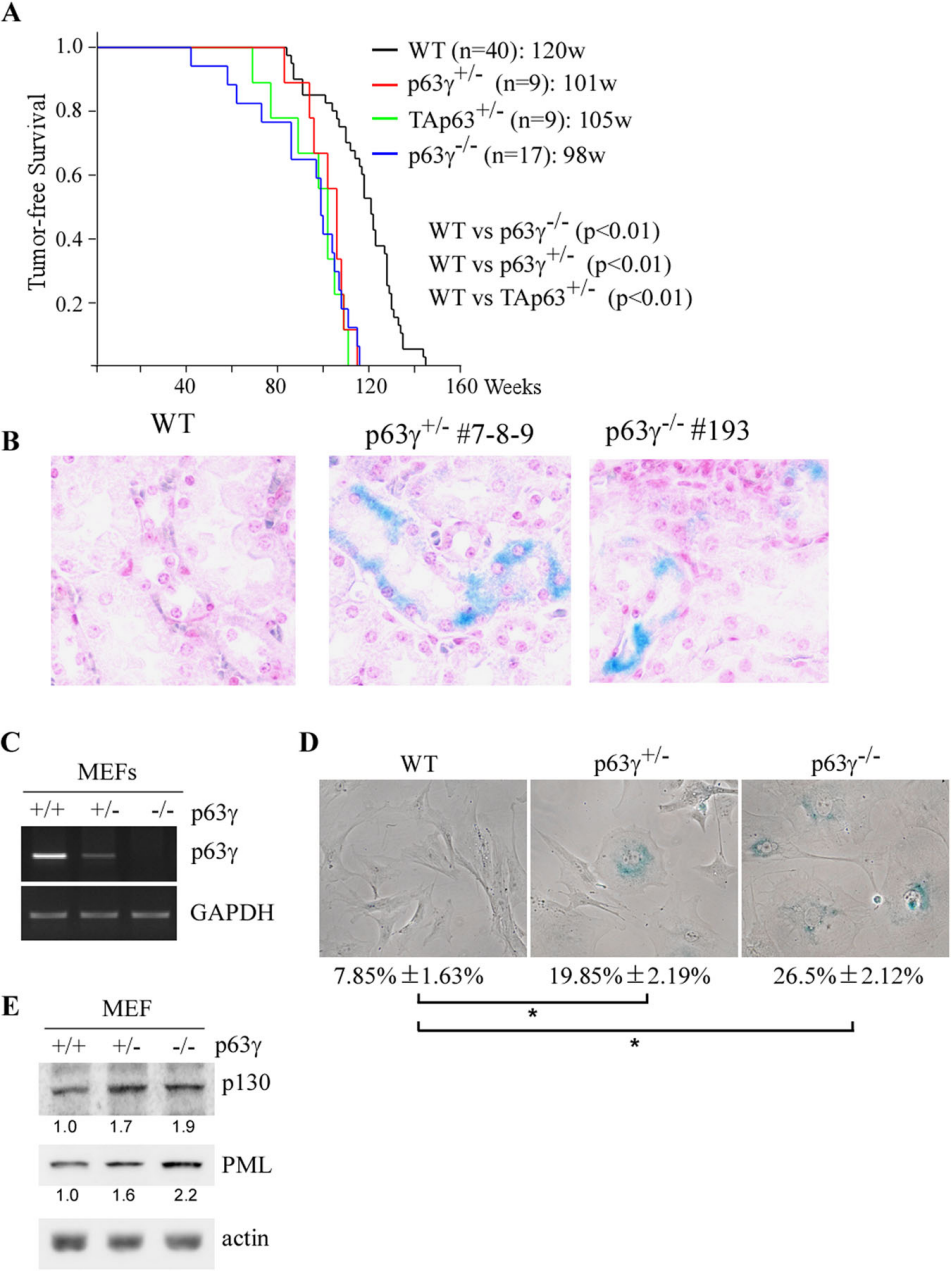
Loss of *p63γ* shortens the lifespan of tumor-free mice and promotes cellular senescence. **A** Kaplan–Meier survival curves of tumor-free WT (*n* = 40), *TAp63*^*+/−*^ (*n* = 9), *p63γ*^*+/−*^ (*n* = 9) and *p63γ*^*−/−*^ (*n* = 17) mice. **B** SA-β-gal assay was performed with kidney tissues from WT, *p63γ*^*+/−*^ and *p63g*^*−/−*^ mice. **C** RT-PCR was performed to measure the levels of p63g and GAPDH transcripts in WT, *p63γ*^*+/−*^ and *p63γ*^*−/−*^ MEFs isolated from 13.5-day-old embryos from the same litter. **D** SA-b-gal assay was performed to measure the percentage of senescence cells from WT, *p63γ*^*+/−*^ and *p63γ*^*−/−*^ MEFs. * indicated *p* < 0.05 by Student’s *t* test. **E** Western blot analysis was performed to measure the levels of p130, PML and actin in WT, *p63γ*^*+/−*^ and *p63γ*^*−/−*^ MEFs used in (**C**). The relative fold change of protein was shown below each lane.

Since ~50% of *p63γ*^*+/−*^ mice (9 of 20) and *p63γ*^−/−^ mice (17 of 31) did not have tumors, their lifespans were compared to tumor-free *TAp63*^*+/−*^ and wild-type mice. We found that like tumor-free *TAp63*^*+/−*^ mice, tumor-free *p63γ*^*+/−*^ and *p63γ*^*−*/−^ mice had much shorter lifespan than tumor-free wild-type mice (Fig. 2A). In addition to tumors, infectious diseases are often the main culprit for early death. However, these mice were housed in the specific pathogen-free environment. Thus, we speculated that premature aging may play a role. To test this, kidney tissues from tumor-free wild-type, *p63γ*-het and *p63γ*-null mice were stained for SA-β-gal, an indicative of premature cellular senescence. We found that both *p63γ*-het and *p63γ*-null mice showed enhanced SA-β-gal staining (Fig. 2B). To verify this, we generated a set of wild-type, *p63γ*-het and *p63γ*-null MEFs from the same litter (Fig. 2C), which were then used for senescence assay. We found that the extent of SA-β-gal-positive MEFs was much higher from *p63γ*-het and *p63γ*^*-*^-null embryos than that from wild-type embryos (Fig. 2D). Moreover, we found that p130 and PML, both of which are used as well-defined senescent markers, were highly expressed in *p63γ*-het and *p63γ*-null MEFs as compared to that in wild-type MEFs (Fig. 2E). These data suggest that loss of p63*γ* promotes cellular senescence, which shortens the lifespan of tumor-free *p63γ*-het and *p63γ*-null mice.

### Mice deficient in *p63*γ are prone to chronic inflammation in multiple organs

**Fig. 3:**
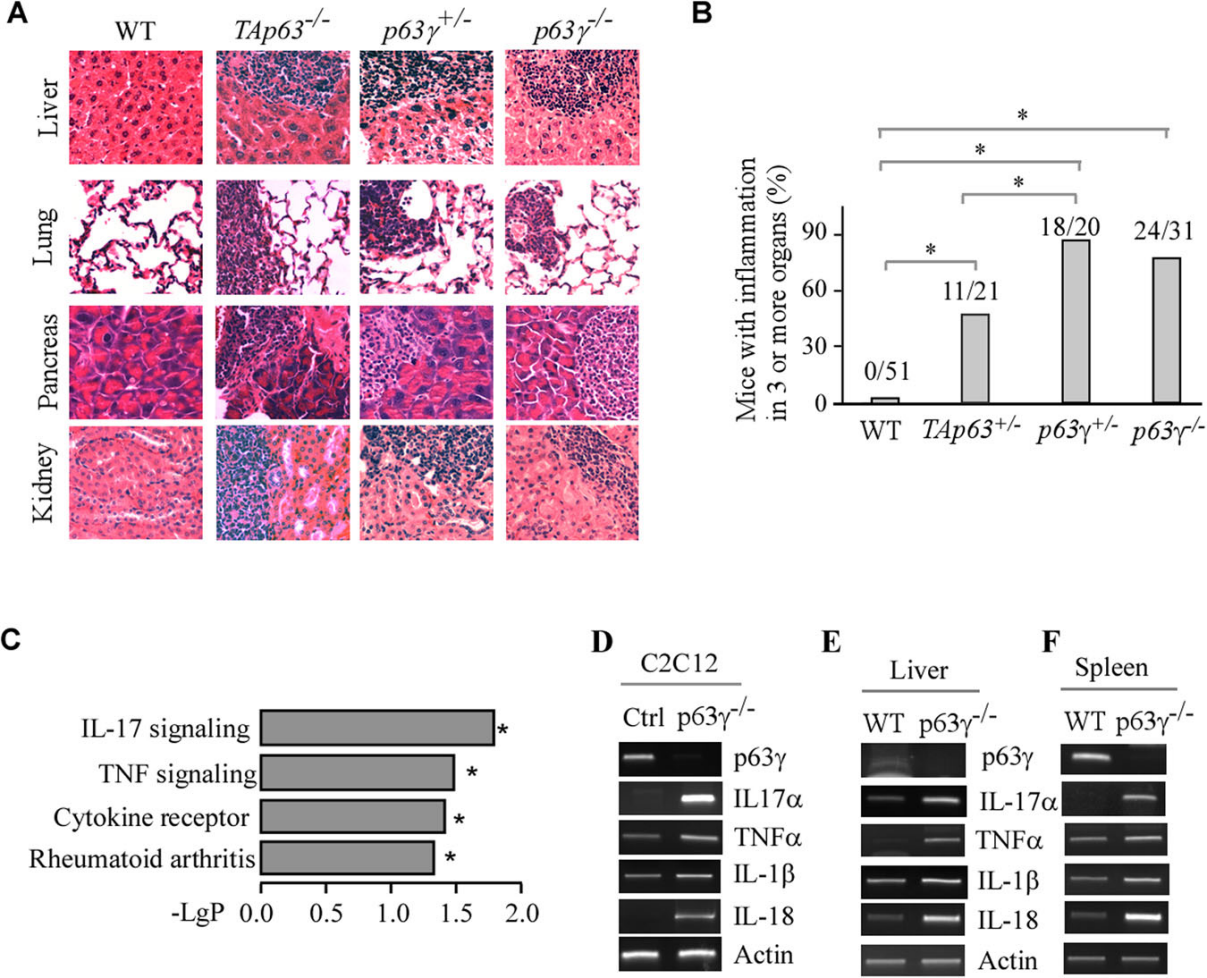
Mice deficient in p63 are prone to chronic inflammation in multiple organs. **A** The percentage of WT, *TAp63*^*+/−*^, *p63γ*^*+/−*^, and *p63γ*^*−/−*^ mice with chronic inflammation in 3 or more organs. **B** Representative images of H&E-stained liver, lung, pancreas, and kidney tissues from WT, *TAp63*^*+/−*^, *p63γ*^*+/−*^, and *p63γ*^*−/−*^ mice. **C** Fold induction of four inflammatory pathways in *p63γ*-KO C2C12 cells as compared to isogenic control cells analyzed by KEGG analyses. **D**–**F** Levels of p63γ, IL17α, TNFα, IL-1β, IL-18, and actin transcripts in isogenic control vs. *p63γ*-KO C2C12 cells (**D**), WT vs. *p63γ*-KO liver (**E**), or WT vs. *p63γ*-KO spleen (**F**).

Histological analysis showed that like *TAp63*^*+/−*^, both *p63γ*^*+/−*^and *p63γ*^*−/−*^ mice exhibited profound chronic inflammation in multiple organs, including liver, lung, pancreas, and kidney (Fig. 3A). Indeed, 18 out of 20 *p63γ*^*+/−*^ mice and 24 out of 31 *p63γ*^*−/−*^ mice whereas none of wild-type mice developed inflammation in three or more organs (Fig. 3B). Statistical analyses indicated that the percentage of chronic inflammation was significantly higher in both *p63γ*^*+/−*^ and *p63γ*^*−/−*^ mice than that of wild-type mice (Fig. 3B). To determine which inflammatory pathway(s) were altered by p63, we generated *p63γ*-KO C2C12 cells using CRISPR-Cas9 method, which were then used for RNA-seq analysis along with isogenic control C2C12 cells. KEGG analyses showed that several inflammatory signaling pathways, including IL-17 and TNF signaling pathways, were significantly increased upon loss of p63γ (Fig. 3C). To verify this, RT-PCR was performed with isogenic control and p63γ-KO C2C12 cells. We found that levels of several cytokine transcripts, including IL17α, TNFα, IL-1β and IL18, were markedly increased by loss of p63γ (Fig. 3D). Furthermore, we found that the levels of transcripts for IL17α, TNFα, IL-1β and IL18 were also increased in the liver and spleen tissues from *p63γ-*KO mice as compared to the ones from wild-type mice (Fig. 3E, F). Together, these data indicated that p63γ plays a critical in inflammatory response by suppressing pro-inflammatory cytokine production.

### Loss of p63γ promotes liver steatosis and alters lipid metabolism

**Fig. 4:**
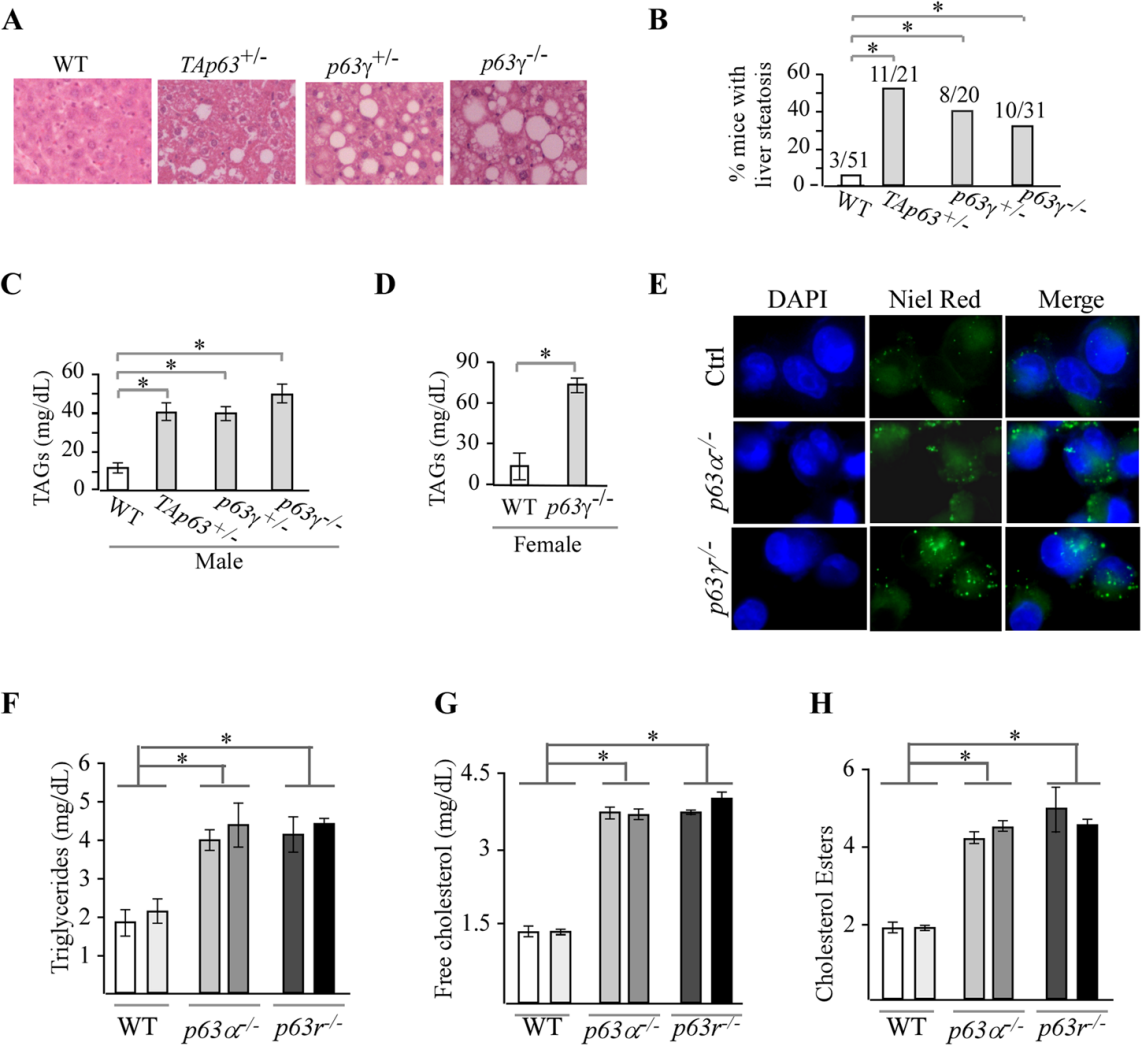
Loss of p63g promotes liver steatosis and alters lipid metabolism. **A** Representative images of H&E-stained livers from 85-week-old WT, *TAp63*^*+/−*^, *p63γ*^*+/−*^, and *p63γ*^*−/−*^ mice. **B** Percentage of WT, *TAp63*^*+/−*^, *p63γ*^*+/−*^, and *p63γ*^*−/−*^ mice with liver steatosis. **C** Serum triglycerides (TAGs) in 16-week-old male mice (3 each genotype). **D** Serum TAGs in 10-week-old female mice (3 each genotype). **E** Representative images of isogenic control, *p63*α^*−/−*^ and *p63γ*^*−/−*^ Mia-PaCa-2 cells stained with DAPI and Nile Red. **F**–**H** TAGs (**F**), free cholesterol (**G**) and CEs (**H**) were measured in two isogenic control, two *p63*α^*−/−*^, and two *p63γ*^*−/−*^ Mia-PaCa-2 cell clones. * indicates *p* < 0.05 by student test.

Previously, we and others showed that *TAp63*-deficient mice are prone to liver steatosis (Fig. 4A, B) [20, 21]. Thus, we examined whether *p63*γ deficiency would make mice prone to liver steatosis. Indeed, we found that like *TAp63*-deficient mice, both *p63γ*^*+/−*^ and *p63γ*^*−/−*^ mice were susceptible to liver steatosis as compared to wild-type mice (Fig. 4A, B). 8 out of 20 *p63γ*^*+/−*^ and 10 out of 31 *p63γ*^*−/−*^ mice developed liver steatosis, which were significantly higher than that of wild-type mice (Fig. 4B). Liver steatosis is a disorder of excess accumulation of lipids, such as triglycerides (TAGs). We thus generated a set of age- and gender-matched mice: three male mice each with the genotype of wild-type, *TAp63*^*+/−*^*, p63γ*^*+/−*^, and *p63γ*^*−/−*^ as well as three wild-type and *p63γ*^*−/−*^ female mice. At 16 weeks of age, sera were collected from these mice and used to measure the level of TAGs. We found that like in *TAp63*^*+/−*^ male mice, TAGs were markedly increased in *p63*γ^*+/−*^ and *p63*γ^*−/−*^ male mice as compared to wild-type mice (Fig. 4C). Similarly, we found that TAGs were elevated in *p63*γ^*−/−*^ female mice as compared to that in wild-type female mice (Fig. 4D). To further test this, Mia-PaCa2 cells, which predominantly express TAp63α/γ [22], were used to generate *p63*α-KO and *p63*γ-KO cells by CRISPR-Cas9. Next, oil red staining was performed to measure the accumulation of lipid droplets in *p63*α-KO and *p63*γ-KO Mia-PaCa2 cells along with isogenic control cells. We found that that the number and size of neutral lipid droplets, which are primarily composed of TAGs and cholesterol esters (CEs), were markedly increased by loss of p63α or p63γ as compared to isogenic control MIA-PaCa-2 cells (Fig. 4E). To further verify this, the levels of intracellular TAGs and CEs were measured by bioluminescent assays [23] in isogenic control, *p63*α-KO and *p63γ*-KO Mia-Paca2 cells. We found that TAGs, free cholesterol and CEs were significantly increased by loss of p63α or p63γ as compared to that in isogenic control cells (Fig. 4F–H). Together, these data suggest that both p63α and p63γ play a critical role in lipid metabolism.

### Single-cell RNA-seq (scRNA-seq) reveals that loss of p63γ increases the population of some immune cells and SCD1 expression

**Fig. 5:**
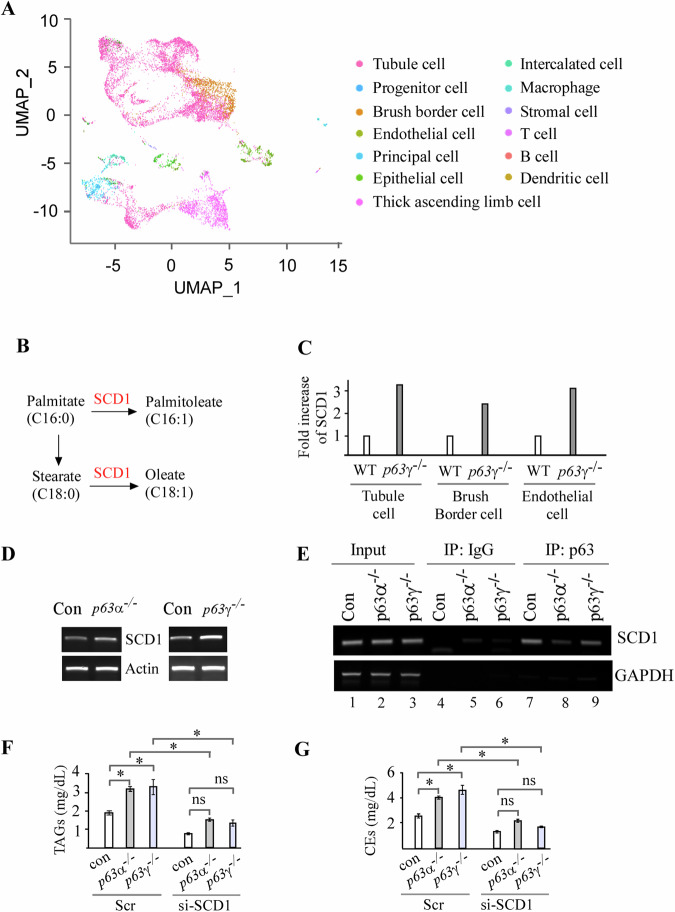
Loss of p63g leads to the accumulation of triglycerides and cholesterol esters via SCD1. **A** UMAP plot of thirteen cell clusters from WT mouse kidneys. **B** Schematic diagram of the functions of SCD1 to converts palmitate (16:0) and stearate (18:0) to palmitoleate (16:1) and oleate (18:1), respectively. **C** SCD1 is induced by loss of p63γ in three cell clusters of mouse kidney cells. **D** SCD1 is induced by loss of either p63α or p63γ in Mia-PaCa2 cells. **E** The level of the SCD1 promoter associated with p63α or p63γ protein was measured by ChIP assay. **F**, **G** The levels of triglyceride (TAG) (**F**) and cholesterol esters (CEs) (**G**) were measured in isogenic control (con), p63α^−/−^ and p63γ^−/−^ Mia-PaCa2 cells transfected with scramble siRNA (Scr) or si-SCD1.

scRNA-seq is a powerful tool to dissect transcriptomic changes in a given cell type from an organ [24]. To understand how p63 regulates transcriptional program associated with lipid metabolism, the 10×Genomics scRNA-seq platform was used to analyze 10-week-old WT and *p63*γ^*−/−*^ mouse kidney cells. 10,864 WT cells and 6967 *p63*γ^*−/−*^ cells were characterized and then segregated into 13 clusters of cells on a Uniform Manifold Approximation and Projection (UMAP) plot [25] (Fig. 5A; Table 2). We noticed that the percentages of T and B cells were doubled in *p63*γ^*−/−*^ kidney (Table 2), which may contribute to the increased inflammation in *p63γ*^*−/−*^ mice (Fig. 3). Most importantly, we found that in some cell types, loss of p63γ led to increased expression of several genes associated with lipid metabolism, especially Stearoyl-CoA desaturase 1 (SCD1). SCD1 converts palmitate (16:0) to palmitoleate (16:1) as well as stearate (18:0) to oleate (18:1), a rate-limiting step in the synthesis of monounsaturated fatty acids (MUFAs) [26, 27] (Fig. 5B). MUFAs are the major fatty acids of TAGs, cholesteryl esters, and membrane phospholipids. Indeed, scRNA-seq showed that SCD1 was induced by loss of p63γ in 3 clusters of kidney cells (Fig. 5C). To examine whether SCD1 is regulated by p63, RT-PCR was performed with isogenic control, p63α-KO, and p63γ-KO Mia-PaCa2 cells. We found that the level of SCD1 transcript was increased by loss of p63α or p63γ in Mia-PaCa2 cells (Fig. 5D). Next, ChIP assay was performed and showed that p63α or p63γ bound to the SCD1 promoter in isogenic control cells, which was markedly decreased in p63α^−/−^ cells (Fig. 5E, compare lane 7 with 8) and to a less extent in p63γ^−/−^ cells (Fig. 5E, compare lane 7 with 9). We speculated that the relative mild reduction of p63 protein bound to the SCD1 promoter in p63γ^−/−^ cells is likely due to the relative high abundance of TAp63α in Mia-PaCa-2 cells. To determine whether SCD1 plays a role in p63-dependent lipid metabolism, we measured the level of TAGs and CEs in isogenic control, *p63*α^*−/−*^ and *p63*γ^*−/−*^ Mia-PaCa-2 cells transfected with a scrambled siRNA or an siRNA against SCD1. We showed that upon transfection of a scrambled siRNA, the levels of TAGs and CEs remained elevated in *p63*α^*−/−*^ and *p63γ*^*−/−*^ cells as compared to isogenic control cells (Fig. 5F, G). However, the levels of TAGs and CEs in *p63*α^*−/−*^ and *p63γ*^*−/−*^ cells were markedly decreased upon knockdown of SCD1 as compared to that upon transfection of a scrambled siRNA (Fig. 5F, G). Moreover, upon knockdown of SCD1, there were no significant differences in the levels of TAGs and CEs among the isogenic control, *p63*α^*−/−*^ and *p63γ*^*−/−*^ cells (Fig. 5F, G). These data suggest that increased expression of SCD1 is required for the accumulation of TAG and CEs in *p63*α^*−/−*^ and *p63γ*^*−/−*^ cells.

**Table 2:** The percentage of each of the thirteen cell clusters identified from WT and *p63γ*-KO kidneys.

Cluster#	Cell type	WT	*p63γ*-KO
1	Tubule cell	64.38%	62.91%
2	Thick ascending limb cell	12.22%	16.57%
3	Brush border cell	10.80%	7.62%
4	Endothelial cell	3.84%	4.12%
5	Principal cell	3.50%	2.83%
6	Epithelial cell	2.98%	3.03%
7	Intercalated cell	1.40%	1.68%
8	Macrophage	0.35%	0.36%
9	Stromal cell	0.24%	0.37%
10	T cell	0.15%	0.32%
11	B cell	0.03%	0.06%
12	Dendritic cell	0.03%	0.04%
13	Progenitor cell	0.06%	0.07%

## Discussion

In this study, we identified TP63γ as the primary isoform of TP63 for tumor suppression but not development by maintaining normal inflammatory response and lipid homeostasis based on the following evidence: (1) *p63γ*-deficeint mice are viable and fertile; (2) *p63γ*-deficeint mice are prone to spontanenous tumors and have a short lifespan; (3) loss of p63*γ* shortens the lifespan of tumor-free mice potentially via increased cellular senescence; (4) *p63γ*-deficeint mice are prone to chronic inflammation; (5) *p63γ*-deficeint mice are prone to liver steatosis and accumulation of serum TAGs and CEs; (6) loss of *p63γ* alters lipid metabolism at least in part via increased expression of SCD1, which promotes the rate-limiting synthesis of monounsaturated fatty acids. Therefore, altered lipid metabolism by elevated expression of SCD1 can be explored as a therapeutic strategy to kill p63*γ*-deficient tumors and chronic inflammation.

It is now evident that *TP63* is not a classic tumor suppressor as *TP53*. Indeed, *TP63* has a more complex and multifaceted role, primarily involving developmental processes, maintaining epithelial integrity, and ensuring genetic quality in germ cells, particularly oocytes. In addition, studies form various p63-deficient mouse models have indicated that the tumor suppressive role of TP63 depends on specific contexts and isoforms, such as TAp63. It should be noted that although TAp63 is suggested to be a tumor suppressor [5], the specific isoform primarily responsible remains unclear. It has been shown that TAp63α isoform plays a crucial role in maintaining genetic integrity and quality control in germ cells, particularly in oocytes [28]. TAp63α is believed to be involved in protecting against DNA damage, preventing the transmission of defective genetic material, and ensuring the proper development of gametes. In line with this, our previous study indicated that among the three TAp63 isoforms, TAp63α is the most abundantly expressed but a relative weaker transcription factor in tumor suppression whereas TAp63β is very active but highly unstable [6], making TAp63γ the most likely candidate for a tumor suppressive role. In support of this, we showed that mice deificient in *p63*γ are prone to spontaneous tumors, suggesting that *TAp63γ* acts as a tumor suppressor. Nevertheless, further studies are needed to further elucidate the role of *TAp63γ* in tumor suppression.

Both altered lipid metabolism and inflammation are hallmarks of cancer. We showed that in *p63γ*-deficeint mice, altered lipid signaling and inflammation are intertwined (Figs. 3–5), which potentially amplifies each other in a pathological setting. Indeed, *p63γ*-deficeint mice are prone to liver steatosis, which would lead to a robust recruitment of immune cells into the liver that in turn produce inflammatory cytokines to propagate the inflammatory response [29]. Mechanistically, we found that loss of p63γ promotes SCD1 expression, which would increase the synthesis of monosaturated fatty acids and subsequently polyunsaturated fatty acids (PUFAs), leading to elevation of leukotrienes and prostaglandins that enhance innate and adaptive immune activity implicated in numerous inflammatory disorders [30, 31]. On the other hand, inflammatory signaling can significantly alter lipid metabolism in the liver and adipose tissue in the context of infection and diabetes. For example, TNFα is found to interfere with lipid homeostasis and activates proatherogenic processes through the SREBP signaling pathway [32]. Importantly, in the tumor microenvironment, cancer cells, infiltrating immune cells and surrounding stromal cells form a well-orchestrated interaction to acquire lipids and produce inflammatory cytokines that promote cell growth and survival [33–35].

We showed that loss of p63γ promotes cellular senescence (Fig. 2). It is well established that cellular senescence is a double-edge sword on lifespan: suppress tumor formation to prolong lifespan but also inhibit cell growth and survival to shorten lifespan. Indeed, a set of p63γ-deficient mice are tumor-free but have a short lifespan. We also showed that tissue senescence is observed in tumor-free *TAp63*^*+/−*^ mice, which is consistent with previous reports [21]. Thus, TAp63γ may play a role in the premature aging observed in TAp63-deficient mice [36].

It is interesting to note that p63γ-deficient mice, which do not express both TAp63γ and ΔNp63γ, do not exhibit any phenotype associated with ΔNp63-deficient mice [37]. One possibility is that ΔNp63α, which is often expressed at a much higher lever than ΔNp63γ, is sufficient to compensate for loss of ΔNp63γ. Another posibility is that ΔNp63γ does not regulate any specific gene necessary for embryonic development and other ΔNp63-associated phenotypes. Nevertheless, further studies are needed to address the underlying mechanism for this phenotype.

## Materials and methods

### Reagents

Anti-Actin (sc-47778, 1:3000), anti- p130 (sc-374521, 1:3000) and anti-PML (sc-377390, 1:3000) were purchased from Santa Cruz Biotechnology. The WesternBright ECL HRP substrate (Cat# K12043-D20) was purchased from Advansta. Nile Red (Cat# N1142) and DAPI (Cat# D3571) were purchased from ThermoFisher Scientific. Scrambled siRNA (5′- GGC CGA UUG UCA AAU AAU U -3′), SCD1 siRNA#1 (5′-GAG AUA AGU UGG AGA CGA U-3′), SCD1 siRNA#2 (5′-GGA GAU AAG UUG GAG ACG A-3′), were purchased from Dharmacon (Chicago, IL). RNAiMax (Life Technologies) was used to transfect siRNA according to the user’s manual. Proteinase inhibitor cocktail was purchased from Sigma-Aldrich. RiboLock RNase Inhibitor and Revert Aid First Strand cDNA Synthesis Kit were purchased from Thermo Fisher. Magnetic Protein A/G beads were purchased from MedChem.

#### Mice and MEF isolation

*TAp63*^*+/−*^ mice were generated as described previously [36]. *p63γ*^*+/−*^ mice were generated by the Mouse Biology Program at University of California at Davis. The genotyping primers for wild-type and *p63γ*^*−/−*^ alleles were a forward primer, 5′ GCT TTT CCG ATT CCC TGC TC 3′ and a reverse primer, 5′ GCA TGT GCA TAT ACA CAA ACG 3′. The PCR products were 313 bp for wild-type allele and 151 bp for *p63γ*^*−/−*^ allele. To isolate WT, *p63γ*^*+/−*^, and *p63γ*^*−/−*^ MEFs, *p63γ*^*+/−*^ mice were intercrossed and mouse embryos at 12.5 to 13.5 postcoitum (p.c.) were used to generate MEFs as described previously [38]. The medium to culture MEFs was DMEM supplemented with 10% FBS (Life Science Technology), 55 μM β-mercaptoethanol, and 1× non-essential amino acids (NEAA) solution (Cellgro).

##### Cell culture and cell line generation

C2C12 and Mia-PaCa2 cells were cultured in DMEM medium supplemented with 10% fetal bovine serum. p63α-KO Mia-PaCa2 and p63γ-KO Mia-PaCa2 cells were generated previously [22]. To generate p63γ-KO C2C12 cells, two sgRNA expression vectors pSpCas9(BB)-2A-Puro-sgp63γ-1 and pSpCas9(BB)-2A-Puro-sgp63γ-2 were transiently transfected into C2C12 cells, followed by puromycin selection for selection. Individual clone was picked and subjected to genotyping to verify the deletion of the p63γ gene. The genotyping primers were a forward primer, 5′-TTT GCT TTG CCT TGC ATC TT-3′ and a reverse primer, 5′-CGT GTT AGT GTT TCC AGC CC-3′.

##### SA-β-Gal assay

The SA-β-Gal assay was performed as described previously [39]. Briefly, 5 × 10^4^ primary MEFs at passage 3 were seeded in a well of 6-well plate for 3 days. Cells were then fixed with fixative solution (2% formaldehyde and 0.2% glutaraldehyde) for 15 min at room temperature, followed by staining overnight at 37 °C with SA-β-galactosidase staining solution (1 mg/mL 5-bromo-4-chloro-3-indolyl-β-d-galactopyranoside, 40 mm citric acid/sodium phosphate (pH 6.0), 5 mm potassium ferrocyanide, 5 mm potassium ferricyanide, 150 mm NaCl, and 2 mm MgCl_2_). The stained cells were then stored in 70% glycerol at 4 °C. The percentage of senescent cells was calculated as SA-β-gal positive cell divided by the total number of cells counted.

##### Western blot analysis

Western blot analysis was performed as previously described [40]. Briefly, whole cell lysates were harvested by 2×SDS sample buffer and boiled at 95 °C for 6 min. Proteins were separated in 8–10% SDS-polyacrylamide gel, transferred to a nitrocellulose membrane, probed with indicated antibodies. To detect protein expression, membrane was incubated with enhanced chemiluminescence and visualized using VisionWorks®LS software (Analytik Jena).

##### RNA isolation and RT-PCR

Total RNA was isolated with Trizol reagent according to user’s manual. cDNA was synthesized with Reverse Transcriptase according to user’s manual. The PCR program used for amplification was (i) 94 °C for 5 min, (ii) 94 °C for 45 s, (iii) 58 °C for 45 s, (iv) 72 °C for 30 s, and (v) 72 °C for 10 min. From steps 2 to 4, the cycle was repeated 22 times for actin and GAPDH, 28–35 times depending on the targets. The primers for mouse GAPDH were a forward primer, 5′-AAC TTT GGC ATT GTG GAA GG-3′ and a reverse primer, 5′-ACA CAT TGG GGG TAG GAA CA-3′. The primers for mouse p63γ were a forward primer, 5′-ATA CAC ACG GAA TCC AGA TG-3′ and a reverse primer, 5′-TTC CTG AAG CAG GCT G AA AG-3′. The primers for mouse IL-1β were a forward primer, 5′-CTC GTG CTG TCG GAC CCA TAT GAG-3′ and a reverse primer, 5′-CTC TGC TTG TGA GGT GCT GAT GTA CC-3′. The primers for mouse IL-18 were a forward primer, 5′-TTG CGT CAA CTT CAA GGA AAT GAT G-3′ and a reverse primer, 5′-CAC AGG CTG TCT TTT GTC AAC GAA G-3′. The primers for mouse IL-17α were a forward primer, 5′-TCT CCA CCG CAA TGA AGA CC-3′, and a reverse primer, 5′-CAC ACC CAC CAG CAT CTT CT-3′. The primers for mouse TNFα were a forward primer, 5′-AGC CCA CGT CGT AGC AAA CCA CCA A-3′ and a reverse primer, 5′-CTA TGC AGT TGA TGA AGA TGT CAA A-3′. The primers for human SCD1 were a forward primer, 5′-TCT ACT TGG AAG ACG ACA TTC GCC C-3′, and a reverse primer, 5′- GGT GGT CAC GAG CCC ATT CAT AGA C-3′.

##### ChIP Assay

ChIP assay was performed as previously described [41, 42]. Briefly, cells were seeded at 1 × 10^7^ per 100-mm plate overnight. Next day, cells were fixed in 1% formaldehyde in phosphate-buffered saline (PBS) to cross-link protein and chromatin. The cells were lysed in RIPA buffer and then sonicated to yield 200- to 1000-bp DNA fragments and immunoprecipitated with a control IgG or p63 antibody. After reverse cross-linking and phenol-chloroform extraction, DNA fragments were purified, followed by PCR to visualize the enriched DNA fragments. The primers to amplify SCD1 promoter were a forward primer, 5′-TGC AGG GGT TTT TCG GAG TTT-3′ and a reverse primer, 5′-TGA ACG CCC TAT TCC AGC CTT A-3′. The primers for GAPDH promoter were a forward primer, 5′-AAA AGC GGG GAG AAA GTA GG-3′, and a reverse primer 5′-AAG AAG ATG CGG CTG ACT GT-3′.

##### Hematoxylin and Eosin staining and histopathological diagnosis

Mouse tissues were fixed in 10% (wt/vol) neutral-buffered formalin, processed, and embedded in paraffin blocks. Tissues blocks were sectioned (6 μm) and stained with hematoxylin and eosin (H&E). The slides were blindly analyzed by a pathologist.

##### Measurement of TAGs and CEs

Cholesterol/Cholesterol Ester-Glo Assay kit (Cat# J3190, Promega) and Triglyceride-Glo Assay kit (Cat# J3160, Promega) from Promega were used to measure Cholesterol and TAGs according to the user’s manual. Briefly, mouse sera or cell lysates were incubated with cholesterol/triglycerides lysis solution at 37 °C for 30 min, and then cholesterol/triglycerides detection reagent for 1 h. The luminescence was then detected using Luminometer (SpectraMAX). The luminescence record of cholesterol, cholesterol Ester, and triglycerides were then converted to abundance information based on a standard curve.

### Nile Red staining

1 × 10^4^ cells were seeded in a slide chamber and then with Nile red in the dark for 20 min at room temperature and counterstained with 4′,6-Diamidino-2-Phenylindole, Dihydrochloride (DAPI). The lipid droplet was then visualized in a confocal microscope (Leica TCS SP8 STED 3X).

### Bulk RNA-seq

Total RNA from WT and p63γ-KO C2C12 cells were isolated and used for bulk RNA-seq analyses. RNA-seq library preparation, sequencing, and sequence data analysis were performed as previously described [43].

### Single-cell RNA-seq

WT and p63γ^−/−^ kidney tissues were used to prepare single cell suspension by using gentleMACS™ Dissociator (Miltenyi Biotec). After removing dead cells with the dead cell remove kit (Miltenyi Biotec), live cells were used for library construction, utilizing the Chromium Next GEM Automated Single Cell 3′ Reagent Kits (10x Genomics, Pleasanton, CA, USA). The libraries were then subjected to next-generation sequencing using an Illumina NovaSeq 6000.

Single-cell RNA sequencing (scRNA-seq) data were processed using Seurat (v4.0.3) after alignment with the Cell Ranger pipeline (v3.1.0) and the GRCm39 reference [44, 45]. Raw gene expression matrices from both wild-type and p63γ^−/−^ samples were imported using the Read10X function. To ensure consistent cell identification across samples, cell barcodes were modified to include sample-specific identifiers. The data were then merged and used to create a Seurat object, retaining cells with at least 200 detected features and genes expressed in at least 10 cells. Mitochondrial gene content was calculated as a percentage of total gene expression, and cell cycle phases were annotated using the cyclone function from the scran package [46]. Data were normalized using a log transformation, and variable features were identified across all genes.

Principal component analysis (PCA) and UMAP were performed to reduce dimensionality and explore clustering, initially without any covariate adjustment. Clusters were identified using a range of resolutions, and UMAP plots were generated for each. Covariates, including UMI count, gene count, and cell cycle phase, were then regressed out, and PCA was rerun. Post-adjustment clustering and dimensionality reduction were visualized using UMAP.

The final Seurat object was used for differential expression analysis between WT and p63γ^−/−^ samples, with UMAP and feature plots generated to visualize key marker genes. This workflow ensures robust analysis and reproducibility, enabling insights into gene expression patterns and cell-type-specific differences between conditions. Data were further explored and analyzed using Shiny app (https://github.com/ucdavis-bioinformatics/scRNA_shiny).

### Statistical analysis

The Log-Rank test was used for Kaplan–Meier survival analysis. Fisher’s exact test or two-tailed Student’s *t* test was performed for the statistical analysis as indicated. *p* < 0.05 was considered significant.

## Supplementary information

Supplemental Figures

Uncut gels

## Data Availability

All study data are included within the article and supplement material.

## References

[1] Yang A, Kaghad M, Wang Y, Gillett E, Fleming MD, Dotsch V, et al. p63, a p53 homolog at 3q27-29, encodes multiple products with transactivating, death-inducing, and dominant-negative activities. Mol Cell. 1998;2:305–16. 9774969 10.1016/s1097-2765(00)80275-0

[2] Mangiulli M, Valletti A, Caratozzolo MF, Tullo A, Sbisa E, Pesole G, et al. Identification and functional characterization of two new transcriptional variants of the human p63 gene. Nucleic Acids Res. 2009;37:6092–104. 19700772 10.1093/nar/gkp674 PMC2764424

[3] Zhang J, Jun Cho S, Chen X. RNPC1, an RNA-binding protein and a target of the p53 family, regulates p63 expression through mRNA stability. Proc Natl Acad Sci USA. 2010;107:9614–9. 20457941 10.1073/pnas.0912594107 PMC2906842

[4] Su X, Napoli M, Abbas HA, Venkatanarayan A, Bui NHB, Coarfa C, et al. TAp63 suppresses mammary tumorigenesis through regulation of the Hippo pathway. Oncogene. 2017;36:2377–93. 27869165 10.1038/onc.2016.388 PMC5415945

[5] Su X, Chakravarti D, Cho MS, Liu L, Gi YJ, Lin YL, et al. TAp63 suppresses metastasis through coordinate regulation of Dicer and miRNAs. Nature. 2010;467:986–90. 20962848 10.1038/nature09459 PMC3055799

[6] Helton ES, Zhang J, Chen X. The proline-rich domain in p63 is necessary for the transcriptional and apoptosis-inducing activities of TAp63. Oncogene. 2008;27:2843–50. 18037962 10.1038/sj.onc.1210948 PMC2662334

[7] Yang A, Schweitzer R, Sun D, Kaghad M, Walker N, Bronson RT, et al. p63 is essential for regenerative proliferation in limb, craniofacial and epithelial development. Nature. 1999;398:714–8. 10227294 10.1038/19539

[8] Mills AA, Zheng B, Wang XJ, Vogel H, Roop DR, Bradley A. p63 is a p53 homologue required for limb and epidermal morphogenesis. Nature. 1999;398:708–13. 10227293 10.1038/19531

[9] Soares E, Zhou H. Master regulatory role of p63 in epidermal development and disease. Cell Mol Life Sci. 2018;75:1179–90. 29103147 10.1007/s00018-017-2701-z PMC5843667

[10] Yan W, Chen X. GPX2, a direct target of p63, inhibits oxidative stress-induced apoptosis in a p53-dependent manner. J Biol Chem. 2006;281:7856–62. 16446369 10.1074/jbc.M512655200

[11] Liao W, Liu H, Zhang Y, Jung JH, Chen J, Su X, et al. Ccdc3: a new P63 target involved in regulation of liver lipid metabolism. Sci Rep. 2017;7:9020. 28827783 10.1038/s41598-017-09228-8 PMC5566403

[12] Boldrup L, Coates PJ, Gu X, Nylander K. DeltaNp63 isoforms differentially regulate gene expression in squamous cell carcinoma: identification of Cox-2 as a novel p63 target. J Pathol. 2009;218:428–36. 19391123 10.1002/path.2560

[13] Mohibi S, Zhang J, Chen X. PABPN1, a Target of p63, Modulates Keratinocyte Differentiation through Regulation of p63alpha mRNA Translation. J Invest Dermatol. 2020;140:2166–77.e6. 32243883 10.1016/j.jid.2020.03.942 PMC7529749

[14] Snaebjornsson MT, Janaki-Raman S, Schulze A. Greasing the wheels of the cancer machine: the role of lipid metabolism in cancer. Cell Metab. 2020;31:62–76. 31813823 10.1016/j.cmet.2019.11.010

[15] Broadfield LA, Pane AA, Talebi A, Swinnen JV, Fendt SM. Lipid metabolism in cancer: new perspectives and emerging mechanisms. Dev Cell. 2021;56:1363–93. 33945792 10.1016/j.devcel.2021.04.013

[16] Bao J, Zhu L, Zhu Q, Su J, Liu M, Huang W. SREBP-1 is an independent prognostic marker and promotes invasion and migration in breast cancer. Oncol Lett. 2016;12:2409–16. 27703522 10.3892/ol.2016.4988 PMC5038874

[17] Mills AA, Zheng BH, Wang XJ, Vogel H, Roop DR, Bradley A. p63is a homologue required for limb and epidermal morphogenesis. Nature. 1999;398:708–13. 10227293 10.1038/19531

[18] Romano RA, Smalley K, Magraw C, Serna VA, Kurita T, Raghavan S, et al. Δ knockout mice reveal its indispensable role as a master regulator of epithelial development and differentiation. Development. 2012;139:772–82. 22274697 10.1242/dev.071191 PMC3265062

[19] Vanbokhoven H, Melino G, Candi E, Declercq W. p63, a Story of Mice and Men. J Investig Dermatol. 2011;131:1196–207. 21471985 10.1038/jid.2011.84

[20] Su X, Gi YJ, Chakravarti D, Chan IL, Zhang A, Xia X, et al. TAp63 is a master transcriptional regulator of lipid and glucose metabolism. Cell Metab. 2012;16:511–25. 23040072 10.1016/j.cmet.2012.09.006 PMC3483083

[21] Jiang Y, Xu E, Zhang J, Chen M, Flores E, Chen X. The Rbm38-p63 feedback loop is critical for tumor suppression and longevity. Oncogene. 2018;37:2863–72. 29520104 10.1038/s41388-018-0176-5 PMC5970038

[22] Yan W, Zhang Y, Chen X. TAp63γ and ΔNp63γ are regulated by RBM38 via mRNA stability and have an opposing function in growth suppression. Oncotarget. 2017;8:78327–39. 29108232 10.18632/oncotarget.18463 PMC5667965

[23] Rojas C, Olivecrona T, Bengtsson-Olivecrona G. Comparison of the action of lipoprotein lipase on triacylglycerols and phospholipids when presented in mixed liposomes or in emulsion droplets. Eur J Biochem. 1991;197:315–21. 2026154 10.1111/j.1432-1033.1991.tb15913.x

[24] Cha J, Lee I. Single-cell network biology for resolving cellular heterogeneity in human diseases. Exp Mol Med. 2020;52:1798–808. 33244151 10.1038/s12276-020-00528-0 PMC8080824

[25] Becht E, McInnes L, Healy J, Dutertre CA, Kwok IWH, Ng LG, et al. Dimensionality reduction for visualizing single-cell data using UMAP. Nat Biotechnol. 2018;37:38–44. 10.1038/nbt.4314 30531897

[26] Igal RA. Stearoyl CoA desaturase-1: New insights into a central regulator of cancer metabolism. Biochim Biophys Acta. 2016;1861:1865–80. 27639967 10.1016/j.bbalip.2016.09.009

[27] Xu H, Luo J, Ma G, Zhang X, Yao D, Li M, et al. Acyl-CoA synthetase short-chain family member 2 (ACSS2) is regulated by SREBP-1 and plays a role in fatty acid synthesis in caprine mammary epithelial cells. J Cell Physiol. 2018;233:1005–16. 28407230 10.1002/jcp.25954

[28] Lena AM, Rossi V, Osterburg S, Smirnov A, Osterburg C, Tuppi M, et al. The p63 C-terminus is essential for murine oocyte integrity. Nat Commun. 2021;12:383. 33452256 10.1038/s41467-020-20669-0 PMC7810856

[29] Koyama Y, Brenner DA. Liver inflammation and fibrosis. J Clin Invest. 2017;127:55–64. 28045404 10.1172/JCI88881 PMC5199698

[30] Lopategi A, Lopez-Vicario C, Alcaraz-Quiles J, Garcia-Alonso V, Rius B, Titos E, et al. Role of bioactive lipid mediators in obese adipose tissue inflammation and endocrine dysfunction. Mol Cell Endocrinol. 2016;419:44–59. 26433072 10.1016/j.mce.2015.09.033

[31] Serhan CN, Chiang N, Dalli J, Levy BD. Lipid mediators in the resolution of inflammation. Cold Spring Harb Perspect Biol. 2014;7:a016311. 25359497 10.1101/cshperspect.a016311 PMC4315926

[32] Tacer Fon, Kuzman K, Seliskar D, Pompon M, Rozman D. D. TNF-alpha interferes with lipid homeostasis and activates acute and proatherogenic processes. Physiol Genomics. 2007;31:216–27. 17566076 10.1152/physiolgenomics.00264.2006

[33] Baumgarten SC, Frasor J. Minireview: Inflammation: an instigator of more aggressive estrogen receptor (ER) positive breast cancers. Mol Endocrinol. 2012;26:360–71. 22301780 10.1210/me.2011-1302 PMC3286192

[34] Incio J, Liu H, Suboj P, Chin SM, Chen IX, Pinter M, et al. Obesity-induced inflammation and desmoplasia promote pancreatic cancer progression and resistance to chemotherapy. Cancer Discov. 2016;6:852–69. 27246539 10.1158/2159-8290.CD-15-1177 PMC4972679

[35] Greten FR, Grivennikov SI. Inflammation and cancer: triggers, mechanisms, and consequences. Immunity. 2019;51:27–41. 31315034 10.1016/j.immuni.2019.06.025 PMC6831096

[36] Su X, Paris M, Gi YJ, Tsai KY, Cho MS, Lin YL, et al. TAp63 prevents premature aging by promoting adult stem cell maintenance. Cell Stem Cell. 2009;5:64–75. 19570515 10.1016/j.stem.2009.04.003 PMC3418222

[37] Romano RA, Smalley K, Magraw C, Serna VA, Kurita T, Raghavan S, et al. DeltaNp63 knockout mice reveal its indispensable role as a master regulator of epithelial development and differentiation. Development. 2012;139:772–82. 22274697 10.1242/dev.071191 PMC3265062

[38] Zhang J, Cho SJ, Shu L, Yan W, Guerrero T, Kent M, et al. Translational repression of p53 by RNPC1, a p53 target overexpressed in lymphomas. Genes Dev. 2011;25:1528–43. 21764855 10.1101/gad.2069311 PMC3143942

[39] Qian Y, Zhang J, Yan B, Chen X. DEC1, a basic helix-loop-helix transcription factor and a novel target gene of the p53 family, mediates p53-dependent premature senescence. J Biol Chem. 2008;283:2896–905. 18025081 10.1074/jbc.M708624200 PMC4118587

[40] Dohn M, Zhang S, Chen X. p63alpha and DeltaNp63alpha can induce cell cycle arrest and apoptosis and differentially regulate p53 target genes. Oncogene. 2001;20:3193–205. 11423969 10.1038/sj.onc.1204427

[41] Harms KL, Chen XB. The c terminus of p53 family proteins is a cell fate determinant. Mol Cell Biol. 2005;25:2014–30. 15713654 10.1128/MCB.25.5.2014-2030.2005 PMC549381

[42] Willis A, Jung EJ, Wakefield T, Chen XB. Mutant p53 exerts a dominant negative effect by preventing wild-type p53 from binding to the promoter of its target genes. Oncogene. 2004;23:2330–8. 14743206 10.1038/sj.onc.1207396

[43] Mohibi S, Zhang J, Chen MY, Chen XB. Mice deficient in the RNA-binding protein Zfp871 are prone to early death and Steatohepatitis in part through the p53-Mdm2 Axis. Mol Cancer Res. 2021;19:1751–62. 34257081 10.1158/1541-7786.MCR-21-0239 PMC8492495

[44] Stuart T, Butler A, Hoffman P, Hafemeister C, Papalexi E, Mauck WM, et al. Comprehensive integration of single-cell data. Cell. 2019;177:1888–902.e21. 31178118 10.1016/j.cell.2019.05.031 PMC6687398

[45] Zheng GX, Terry JM, Belgrader P, Ryvkin P, Bent ZW, Wilson R, et al. Massively parallel digital transcriptional profiling of single cells. Nat Commun. 2017;8:14049. 28091601 10.1038/ncomms14049 PMC5241818

[46] Scialdone A, Natarajan KN, Saraiva LR, Proserpio V, Teichmann SA, Stegle O, et al. Computational assignment of cell-cycle stage from single-cell transcriptome data. Methods. 2015;85:54–61. 26142758 10.1016/j.ymeth.2015.06.021

