# Supplementary material for: TAp63γ is the primary isoform of TP63 for tumor suppression but not development
Source: Cell Death Discov. 2025 Feb 6;11:51. doi: 10.1038/s41420-025-02326-x (PMC11802870; doi:10.1038/s41420-025-02326-x)
Supplement: Supplementary file 1 — Supplemental Figures [file 41420_2025_2326_MOESM1_ESM.pdf]

Supplemental Table S1: Wild-type (WT) mice (n=56) - survival time, tumor spectrum, steatosis, inflammation, and other abnormalities

ID	Gender	Survival (Wks)	Tumor	Steatosis	inflammation	Other abnormalities
5	F	134	-	-	-	-
7	F	117	-	-	-	-
16	F	100	-	-	-	-
22	F	109	-	-	-	-
25	F	109	-	-	-	-
44	F	90	-	-	-	-
55	F	104	T-LBL	-	-	-
64	F	120	-	-	-	-
2	M	127	-	-	-	-
3	M	117	-	-	Liver	-
12	M	127	-	-	-	-
13	M	127	-	-	-	-
20	M	122	-	-	-	-
23	M	122	-	-	-	-
26	M	127	-	-	Liver/Salivary gland	-
34	M	124	DLBCL	-	-	-
37	M	134	-	-	-	-
62	M	128	-	-	-	-
45	M	133	-	-	-	-
49	M	117	-	-	-	-
50	M	113	T-LBL/ DLBCL	-	-	-
56	M	117	DLBCL	-	-	-
59	M	119	DLBCL	-	-	-
65	M	106	-	-	-	-
69	M	102	DLBCL	-	-	SH
70	M	103	-	-	-	TH
71	M	90	-	-	-	-
1-24-2	M	83	-	Yes	-	-
2-15-2	F	140	Lymphoma	-	-	EMH in liver
2-19-6	M	132	-	-	Pancreas	EMH in Spleen
2-19-2	F	143	-	-	-	EMH in Spleen
3-11-3	M	129	-	-	-	-
3-28-5	F	85	-	Yes	-	EMH in spleen
3-9-7	M	129	-	-	-	-
5-12-3	F	99	Lymphoma	-	-	EMH in Spleen
7-9-9	F	116	-	-	-	-
8-2-6	M	120	-	-	-	-
10-24-7	F	130	-	-	Pancreas	EMH in spleen
10-26-6	F	129	Histiocytic sarcoma	-	-	EMH in spleen/liver
11-10-7	M	121	-	-	-	-
11-7-3	F	121	-	-	-	-
11-29-2	F	144	-	Yes	-	EMH in spleen
12-2-4	F	113	-	-	-	-
12-20-7	F	115	-	-	-	EMH in Spleen
11-9-6	F	96	DLBCL	-	Skin	-
1-19-1	F	86	-	-	Skin	-
11-10-15	F	105	-	-	Skin/Pancreas	-
12-25-6	M	111	-	-	-	Hepatocirrhosis
1-19-5	F	109	Lymphoma	-	-	-
7-22-4	M	86	-	-	-	-
7-22-7	M	126	-	-	Kidney	-
11	M	111	N/A			Found dead
42	M	111	N/A			Found dead
43	M	107	N/A			Found dead
46	M	117	N/A			Found dead
52	M	101	N/A			Found dead

These mice were from published studies (Yang et al, 2017, PNAS, 114 (43) 11500-11505; Zhang et al, 2017, Genes & Dev, 31:1243-56)
DLBCL: Diffuse Large B-cell Lymphoma; EMH: Extramedullary Hematopoiesis; TH: Thymic Hyperplasia; SH: Splenic Hyperplasia
N/A: not applicable

Supplemental Table S2: Survival time and tumor spectrum in *TAp63*$^{+/-}$ mice (n=21)

ID	Gender	Survival Time (W)	Tumor	Liver Steatosis	Inflammation	Other abnormalities
11	M	78	Hemangioma	Yes	-	-
13	M	121	Angiosarcoma	Yes	Kidney; Liver	EMH
14	M	99	Lymphoma; angiosarcoma	-	Kidney; Liver; Lung; Pancreas; salivary gland	EMH
16	M	124	Angiosarcoma	-	Kidney; Liver; Pancreas	SWPH
17	F	103	B-cell lymphoma; angiosarcoma	-	Lung; Pancreas; Salivary gland; Kidney	-
25	M	118	Angiosarcoma	-	Kidney; Liver; Pancreas	SRPH
28	M	95	Liposarcoma	Yes	Kidney; Liver; Lung; Pancreas	-
5	F	108	Lymphoma	-	Liver; Lung; Salivary gland;Kidney	-
3	F	122	Lymphoma	Yes	Liver; Lung; Pancreas;Kidney	SPWH
10-2-1	M	91	Angiosarcoma	-	-	Red pulp hemorrhage
11-15-2	M	83	Lymphoma	Yes	-	Red pulp hemorrhage
1-20-1	F	75	Hemangiosarcoma	Yes	Kidney; Lung	SPWH, Glomerulosclerosis
19	F	97	-	Yes	Liver; Pancreas; Salivary gland;kidney	-
10	M	88	-	-	Lung;kidney	-
10-2-4	M	110	-	-	Liver; Salivary Gland	Red pulp hemorrhage
3-2-15	F	108	-	Yes	Kidney; Pancreas; Salivary gland	-
21	M	101	-	Yes	Kidney; Lung; Salivary gland	EMH, SPWH
11-15-3	M	104	-	-	Liver; Lung; Pancreas; Salivary gland	Glomerulosclerosis
11-19-1	M	76	-	Yes	-	-
2-13-6	F	68	-	Yes	-	-
27	M	101	-	-	-	-

These mice were from published studies (Jiang et al, 2018, Oncogene, 37 (21) 2863-2872.

EMH extramedullary hematopoiesis; SWPH: Spleen white pulp hyperplasia; SRPH: Spleen red pulp hyperplasia

Supplemental Table S3: *p63r* $^{+/-}$ mice (n=20) - survival time, tumor spectrum, inflammation, and other abnormalities

ID	Gender	Survival (Wks)	Tumor	Steatosis	Inflammation	Other abnormalities
4	M	82	-	Yes	Kidney; Salivary gland;skin	SWPH/EMH/TH
44	M	93	-	Yes	Lung; Kidney; Salivary gland	SWPH/EMH/TH
11	F	89	large B cell lymphoma	-	Kidney	-
41	F	93	large B cell lymphoma	-	Liver; Lung; Kidney	TH
31	M	94	HCC	-	Liver; Lung; Kidney;Salivary gland; skin	-
8	M	97	sebaceous adenoma	-	Liver; Lung; Kidney; Salivary gland	SWPH/EMH/TH
13	M	105	-	-	Liver; Lung; Kidney; Salivary gland	SWPH/EMH/TH
2	M	107	-	-	Liver; Lung; Kidney; Salivary gland	SWPH/EMH/TH
34	M	105	-	Yes	Lung; Kidney; Salivary gland	SWPH/EMH/TH
16	M	109	spindle cell sarcoma	-	Liver; Lung; Kidney; Salivary gland	SWPH/EMH
49	F	108	-	-	Liver; Lung; Kidney; Salivary gland; pancreas; intestine	SWPH/EMH/TH
26	F	114	Papillary adenocarcinoma	-	Liver; Kidney; Salivary gland	SWPH/EMH/TH
28	F	114	-	Yes	Liver; Lung; Kidney; Salivary gland	SWPH/EMH/TH
27	F	120	large B cell lymphoma	Yes	Lung; Kidney; Salivary gland/liver	EMH/TH
47	F	113	large B cell lymphoma	Yes	Lung; Kidney; Salivary gland/liver	EMH/TH
36	F	67	infarcted hematoma	Yes	Lung; Kidney; Salivary gland/liver	EMH/TH
7-18-2	F	95	-	Yes	Lung; Kidney; Salivary gland/liver	EMH/TH
7-18-9	F	101	-	-	Lung; Kidney; Salivary gland/liver	SWPH/EMH/TH
7-18-6	F	101	large B cell lymphoma	-	Lung; Kidney; Salivary gland/liver	EMH
32	M	72	large B cell lymphoma	-	pancreas/lung	benign cyst in kidney

SWPH: Spleen white pulp hyperplasia; EMH: extramedullary hematopoiesis; TH: Thymus hyperplasia;

Supplemental Table S4: *p63r*$^{-/-}$ mice (n=31) - survival time, tumor spectrum, inflammation, and other abnormalities

ID	Gender	Survival (Wks)	Tumor	Steatosis	Inflammation	Other abnormalities
63	F	103	Hemangioma; Sarcoma	-	kidney; salivary gland	EMH/TH
73	M	91	HCC	Yes	kidney; salivary gland	SWPH
80	M	92	B-cell lymphoma	-	Liver; salivary gland	EMH
91	F	84	B-cell lymphoma	-	Liver; salivary gland	EMH
93	F	84	B-cell lymphoma	-	Liver; salivary gland	EMH
111	M	85	-	-	Liver; salivary gland;kidney	SWPH/EMH
131	F	104	-	-	Liver; salivary gland;kidney	SWPH/EMH
142	F	72	-	-	Liver; salivary gland;kidney	SWPH/EMH/TH
145	F	57	-	-	Liver; salivary gland;kidney;ovary	SWPH/EMH/TH
189	F	85	-	-	Liver; salivary gland;kidney;pancreas	SWPH/EMH/TH
190	F	41	-	-	Liver; salivary gland;kidney;ovary	SWPH/EMH/TH
121	M	98	-	-	Liver; salivary gland;kidney	SWPH/EMH/TH
123	F	61	-	-	Liver; salivary gland;kidney	EMH
128	F	116	HCC; Hemangiosarcoma; Lymphoma	Yes	Liver; salivary gland;kidney;lung	-
143	M	111	B-cell lymphoma	-	Liver; salivary gland;kidney	SWPH/EMH
152	F	113	MALT-Lymphoma	Yes	Liver; salivary gland;kidney;lung	SWPH/EMH
156	M	110	B-cell lymphoma	-	salivary gland	SWPH/EMH
160	F	110	B-cell lymphoma	Yes	Liver; salivary gland;kidney; pancreas	SWPH/EMH/TH
163	F	122	B-cell lymphoma	Yes	Liver; salivary gland;kidney	SWPH/EMH/TH
166	M	110	-	-	Liver; salivary gland;kidney	SWPH/EMH/TH
171	F	106	-	Yes	Liver; salivary gland;kidney	SWPH/EMH
180	F	115	-	-	Liver; salivary gland;kidney	SWPH/EMH
182	F	80	B-cell lymphoma	-	Liver; salivary gland;kidney	SWPH/EMH
185	F	108	B-cell lymphoma	-	Liver; salivary gland;kidney;Lung	SWPH/EMH/TH
188	F	96	-	-	Salivary gland;kidney;Lung	SWPH/EMH/TH
193	F	99	-	Yes	Salivary gland;kidney;Lung	SWPH/EMH
196	M	103	-	Yes	Liver; salivary gland;kidney	SWPH/EMH
69	M	107	-	-	Liver; salivary gland;kidney	SWPH/EMH
75	F	98	-	Yes	Liver; salivary gland;kidney;Lung	SWPH/EMH/TH
74	F	113	Lung adenocarcinoma	Yes	Liver; salivary gland;kidney	SWPH/EMH
87	M	114	-	-	salivary gland;kidney	SWPH/EMH

SWPH: Spleen white pulp hyperplasia; EMH: extramedullary hematopoiesis; TH: Thymus hyperplasia;
