# Supplementary figures and images for: TAp63γ is the primary isoform of TP63 for tumor suppression but not development
Source: Cell Death Discov. 2025 Feb 6;11:51. doi: 10.1038/s41420-025-02326-x (PMC11802870; doi:10.1038/s41420-025-02326-x)

Fig. 1D

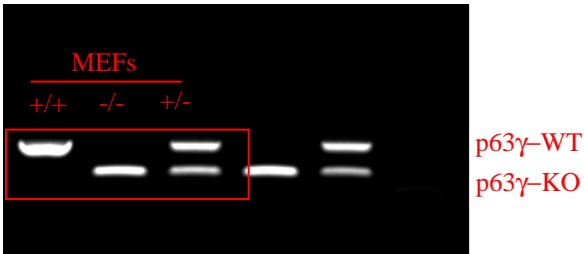

Fig. 2C

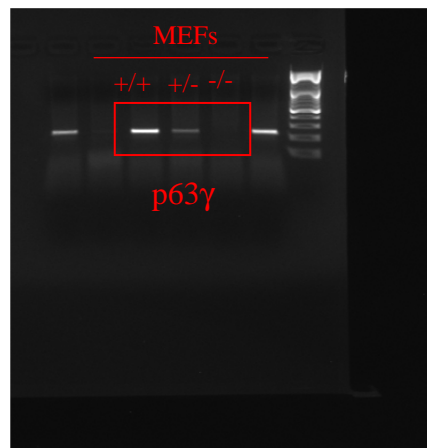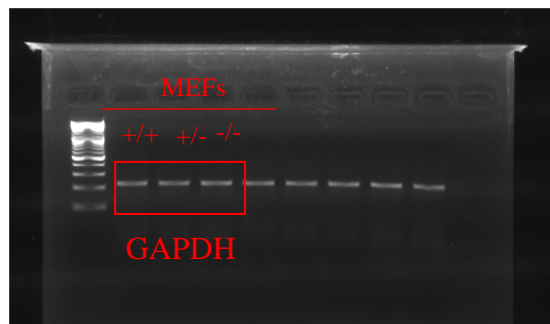

Fig. 2E

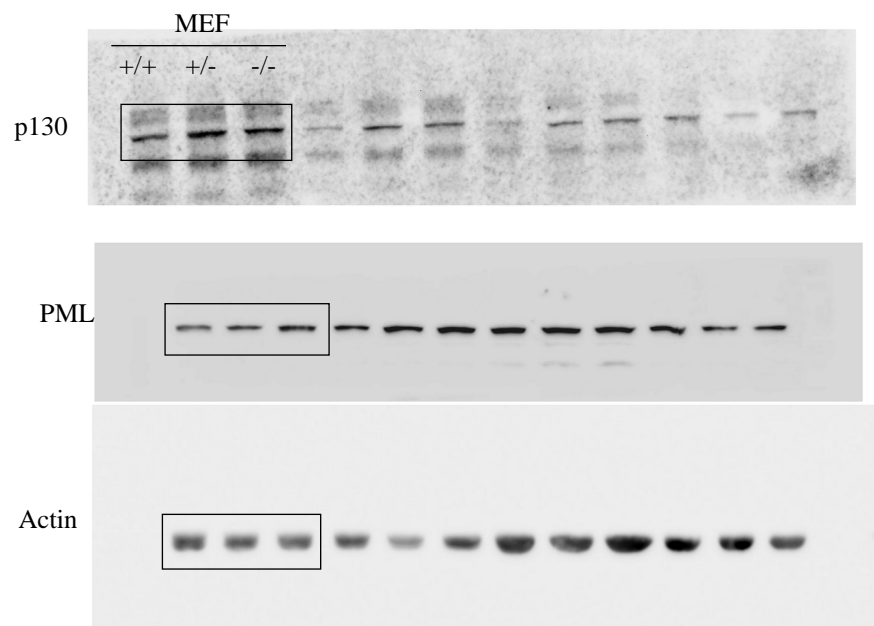

Fig. 3D

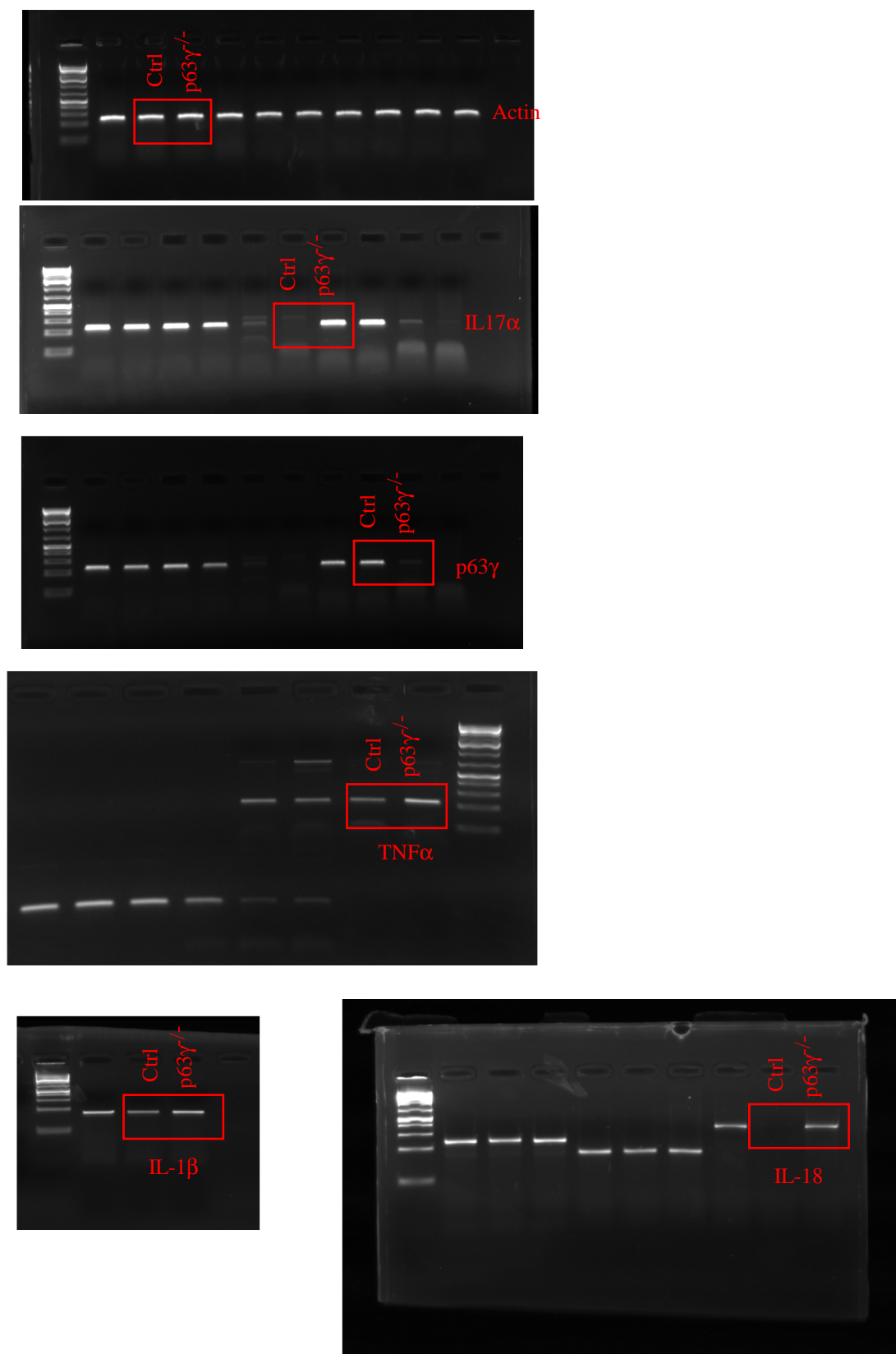

**Fig 3E**

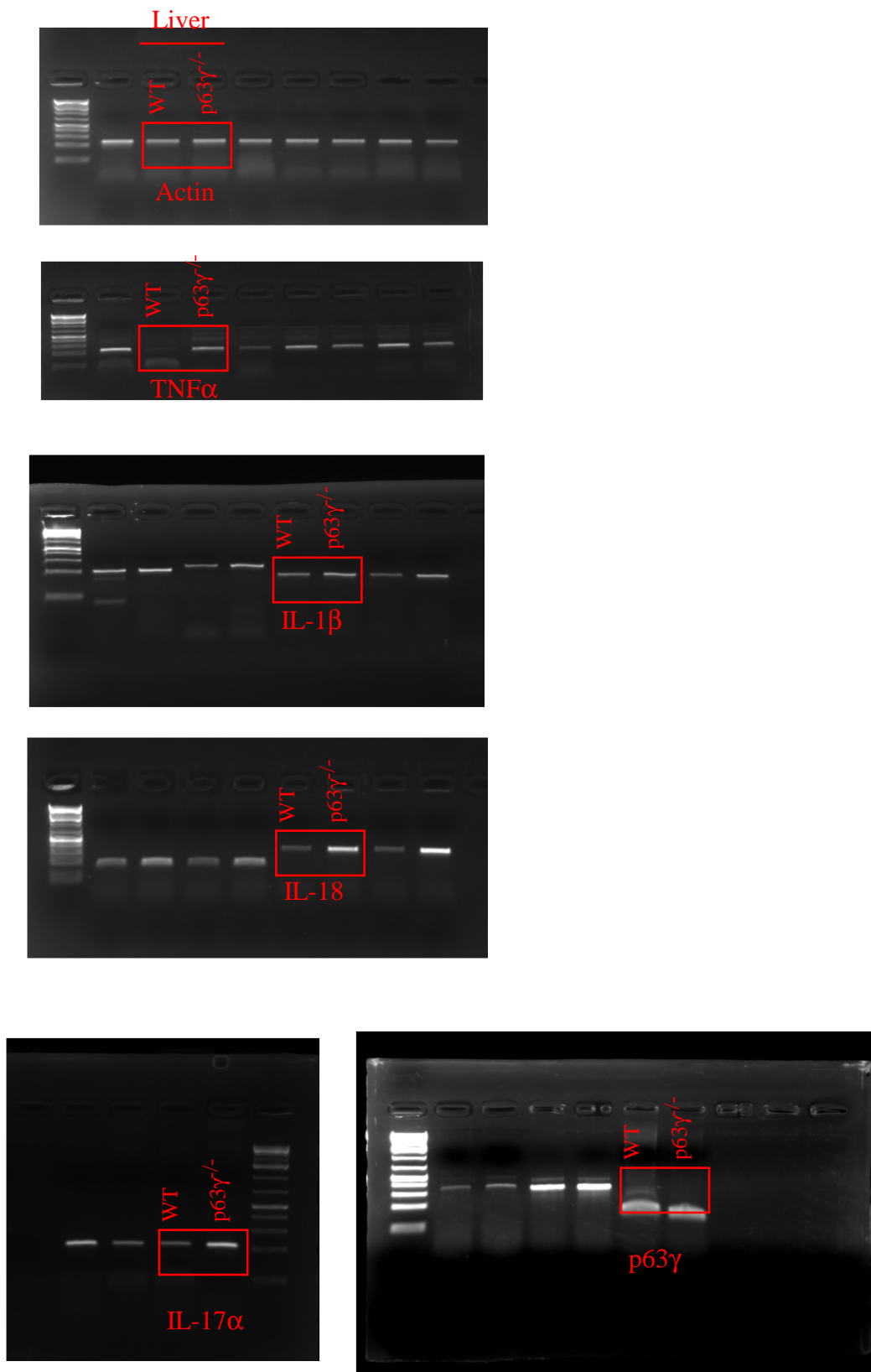

**Fig. 3F**

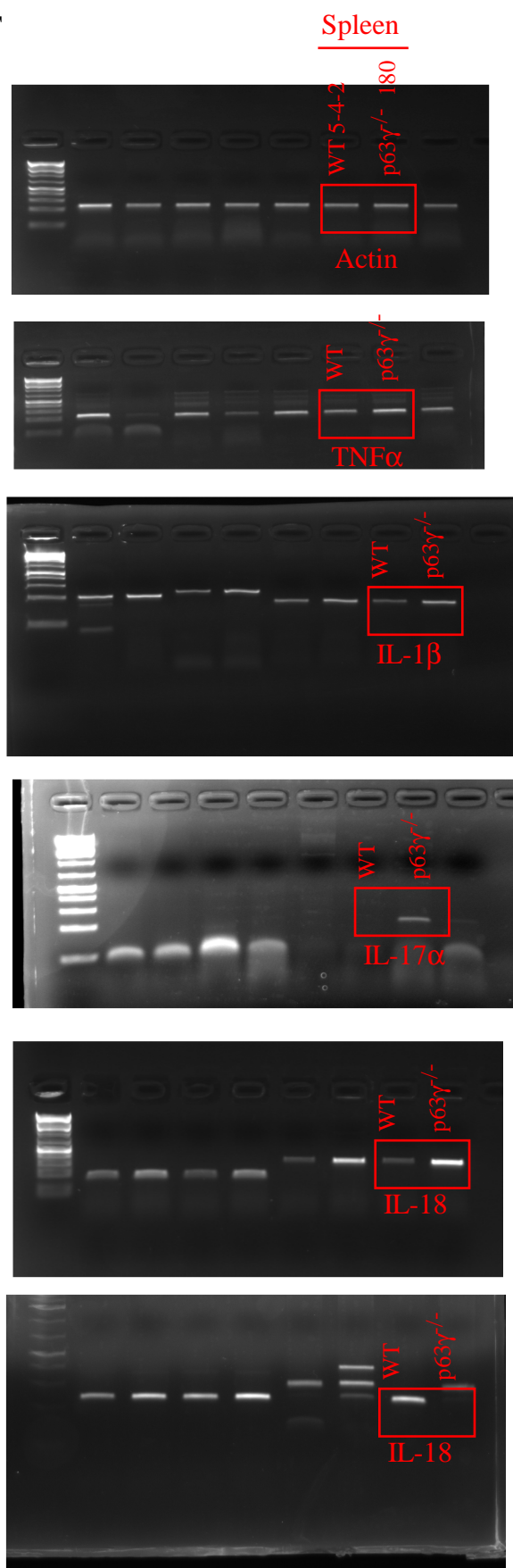

**Fig. 5E**

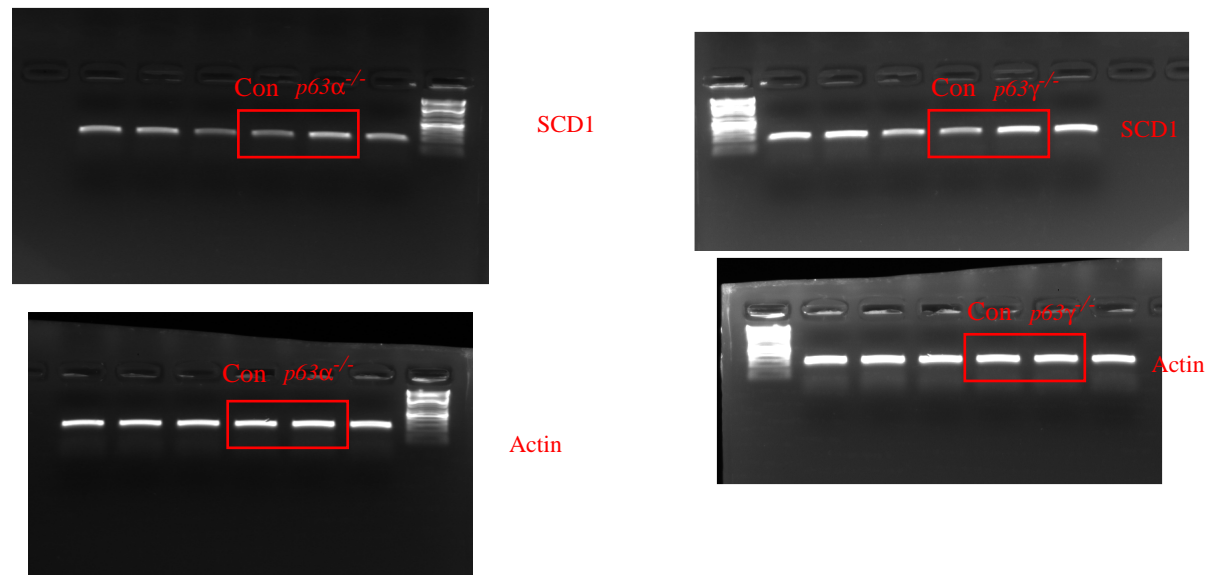

**Fig 5F**

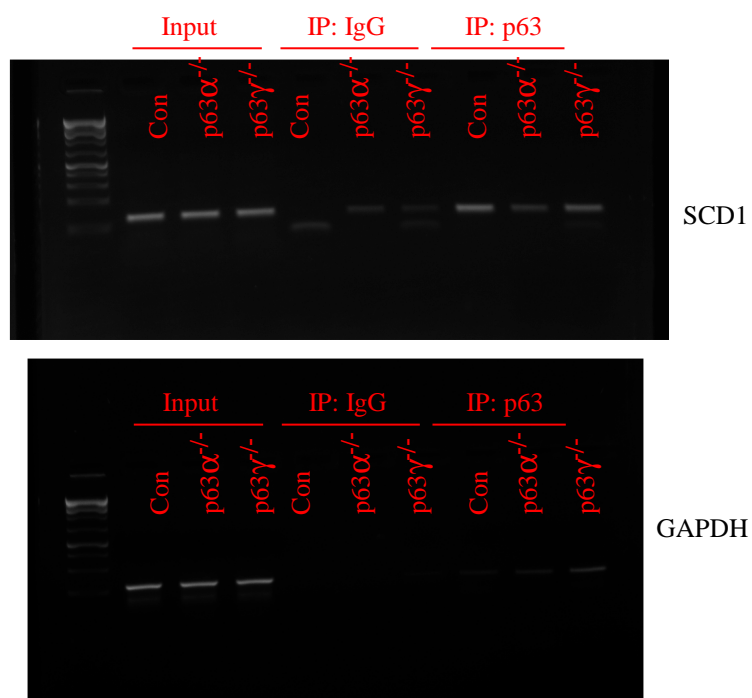

Supplement: Supplementary file 2 — Uncut gels [file 41420_2025_2326_MOESM2_ESM.pdf]
